# Mechanical strength of stock and custom abutments as original and aftermarket components after thermomechanical aging

**DOI:** 10.1002/cre2.892

**Published:** 2024-07-25

**Authors:** Christiaan W. P. Pol, Marco S. Cune, Gerry M. Raghoebar, Lucas Z. Naves, Henny J. A. Meijer

**Affiliations:** ^1^ Department of Integrated Dentistry, Dental School, University Medical Center Groningen University of Groningen Groningen The Netherlands; ^2^ Department of Restorative Dentistry, Dental School, University Medical Center Groningen University of Groningen Groningen The Netherlands; ^3^ Department of Oral and Maxillofacial Surgery Prosthodontics and Special Dental Care, St. Antonius hospital Nieuwegein Nieuwegein The Netherlands; ^4^ Department of Oral and Maxillofacial Surgery, University Medical Center Groningen University of Groningen Groningen The Netherlands

**Keywords:** dental implant‐abutment designs, dental implants, materials testing, nonoriginal components

## Abstract

**Objectives:**

The study aimed to assess the impact on the mechanical strength and failure patterns of implant‐abutment complexes of choosing different abutment types, designs and manufacturers, aiding in selecting the optimal restorative solution. Stock and custom abutments from original and aftermarket suppliers were subjected to thermomechanical aging.

**Material and Methods:**

Stock and custom abutments from the implant manufacturer (original) and a aftermarket supplier (nonoriginal) were connected to identical implants with internal connection. Custom abutments were designed in a typical molar and premolar design, manufactured using the workflow from the respective suppliers. A total of 90 implants (4 mm diameter, 3.4 mm platform, 13 mm length) equally divided across 6 groups (three designs, two manufacturers) underwent thermo‐mechanical aging according to three different regimes, simulating five (*n* = 30) or 10 years (*n* = 30) of clinical function, or unaged control (*n* = 30). Subsequently, all samples were tested to failure.

**Results:**

During aging, no failures occurred. The mean strength at failure was 1009N ± 171, showing significant differences between original and nonoriginal abutments overall (−230N ± 27.1, *p* < .001), and within each abutment type (*p* = .000), favoring original abutments. Aging did not significantly affect the failure load, while the type of abutment and manufacturer did, favoring original and custom‐designed abutments. The most common failure was implant bending or deformation, significantly differing between original and nonoriginal abutments and screws. All failure tests resulted in clinically unsalvageable implants and abutments.

**Conclusions:**

Within the limitations of this study, original abutments exhibited a higher mechanical strength compared to the nonoriginal alternative, regardless of the amount of simulated clinical use. Similarly, custom abutments showed higher mechanical strength compared to stock abutments. However, mechanical strength in all abutments tested was higher than average chewing forces reported in literature, thus components tested in this study can be expected to perform equally well in clinical situations without excessive force.

## 1 INTRODUCTION

It is recommended to plan ahead in any treatment, but especially in one as costly, lengthy and invasive as implant treatment (Joda et al., 2018). To achieve the desired outcome, it is necessary to consider the technical and practical possibilities in relation to the patient's needs, desires, and limitations (Ramani et al., 2020). Each choice in implant treatment has consequences that can affect various aspects, including mechanical strength, gingival health, esthetics, and cost (Pol et al., 2018). By carefully weighing the available options, the dentist can help the patient in achieving the best possible result in all aspects (Lang et al., 2012).

For patients who require treatment for a missing tooth, implants have become a popular choice (Sanz et al., 2019). However, after selecting an implant‐based reconstruction, numerous choices still need to be made (Rizvi et al., 2022). Cost remains a concern for many patients, and one way to reduce the financial burden is to use less expensive components (Alonso‐Pérez et al., 2022).

Over the years, several companies have started offering nonoriginal prosthetic components, such as abutments and screws, to restore implants from different manufacturers (Rizvi et al., 2022). These components are marketed to either reduce the cost compared to original components or to offer alternative prosthetic solutions not available from the implant manufacturer (Alonso‐Pérez et al., 2022; Rizvi et al., 2022; Tallarico et al., 2018). Next to this, technological advancements have allowed for a diverse range of customized components and digital design and manufacturing techniques have further enabled the customization of components (Apicella et al., 2010; Hsu et al., 2019; Raee et al., 2021; Sanz et al., 2019). Customized abutments have esthetic benefits over stock abutments and excess cement is more easily removed, but these abutments are more expensive. Especially in less challenging or demanding situations stock abutments can be used (Zarauz et al., 2020).

The choices made in the type and manufacturer of implant components used in a treatment can impact various treatment outcomes, though should ideally not lead to a result that is significantly less durable (Pjetursson et al., 2018, 2021). For various reasons, aftermarket suppliers often make changes to the design and the materials used compared to the original components, but do not regularly report on the effect of the mechanical strength this alteration might have (Jarman et al., 2017). Scientific research and testing can provide guidance in making treatment planning decisions. Still, the abundance of materials available makes it difficult to test all possible combinations in controlled clinical trials (Coray et al., 2016).

In vitro research can be a useful tool in dental implant research. Specifically, in vitro testing allows for a controlled environment that can help eliminate confounding variables and better isolate the effects of implant components on specific outcomes, such as mechanical properties. In addition, in vitro research can be conducted more rapidly and at a lower cost than clinical studies. This allows for a larger number of standardized implant components to be tested and evaluated. Furthermore, in vitro testing can be used to identify potential issues with implant components before they are tested in clinical studies, potentially saving time and resources—not to mention the burden on patients—by avoiding costly clinical trials that may be less likely to succeed (Mühlemann et al., 2014; Sailer et al., 2018).

Several in vitro testing methods have been proposed to evaluate the clinical performance of various implant abutment systems (Alonso‐Pérez et al., 2022; Coray et al., 2016; Rizvi et al., 2022). However, some of these methods, including gap formation assessment, leakage evaluation, and preload measurement, lack sensitivity or are heavily influenced by confounding factors. Thermomechanical aging is considered a reliable and feasible method to assess the overall performance of abutment systems (Karl & Irastorza‐Landa, 2018). Laboratory studies, which use chewing simulators and thermocycling, can simulate an amount of aging in a matter of days that would take several years to occur in vivo. Thus, artificial aging of dental materials is a useful tool to predict how these materials will perform in clinical settings, as demonstrated by previous studies on various implant abutment concepts (Coray et al., 2016; Mahmoud, 2012; Mühlemann et al., 2014; Sailer et al., 2018; Steinebrunner et al., 2008).

The aim of this study was to determine the effect of selecting stock or custom abutments from original or nonoriginal suppliers on the mechanical strength and failure modes of the implant‐abutment complex, aiding in selecting the optimal restorative solution. Effects were determined using an in vitro testing methods with thermomechanical aging, based on protocols and previous literature. The hypothesis was that using nonoriginal or stock abutments has no significant negative impact on the mechanical strength of the implant‐abutment complex and can thus be done in all clinical situations.

## 2 MATERIALS AND METHODS

Dental implants from a single type and manufacturer were outfitted with titanium abutments in three different abutment types obtained from both the original and a nonoriginal manufacturer. Two groups consisted of a patient‐specific abutment, in a molar and a premolar design, the other type was a stock abutment. Each group comprised 15 identical implant‐abutment assemblies of both manufacturers, totaling 90 specimens and resulting in six distinct groups. All implants, abutments and screws were standard components procured via routine ordering procedures and arrived complete with packaging and lot numbers. Table 1 outlines the materials utilized in this study.

**Table 1:** Overview of implant components used in the study.

Implant system	ZimVie T3 with DCD, 3.4 mm connection, 4 mm platform, 13 mm length, model BNPT4313
Nonoriginal custom abutments	Medentika, PreFace abutment for Biomet 3i Certain, 3.4 mm diameter, model H‐9000‐R
Original custom abutments	ZimVie, BellaTek, CAD/CAM definitive abutments, model 3MAT3
Nonoriginal stock abutment	Medentika, engaging titanium base abutment for Biomet 3i Certain, 3.4 mm diameter, 5.5 mm height, model H‐1100
Original stock abutment	ZimVie, FlexLink TiBase, 3.4 mm diameter, 5.5 mm height, engaging, model IEMTB51G

### 2.1 Implant preparation

Implants (*n* = 90) with an internal hexagonal connection, a length of 13 mm, diameter of 4 mm and a connection of 3.4 mm (ZimVie T3 with DCD, ZimVie Dental, Palm Beach Gardens) were used. All implants were accurately embedded in cylinders made of phenolic resin (Buehler Ltd.) with 3 mm of simulated bone loss as recommended by ISO 14801 (International Organization for Standardization, 2016).

### 2.2 Abutment preparation

Half of the implants were fitted with implant‐specific abutments (custom BellaTek 3MAT3 or stock FlexLink TiBase IEMTB51G from ZimVie Dental), while the other half were fitted with the corresponding component from a third‐party supplier (patient‐specific H‐9000‐R or stock H‐1100 from Medentika). The connection screw packaged with each specific abutment was used for each sample.

**Figure 1:**
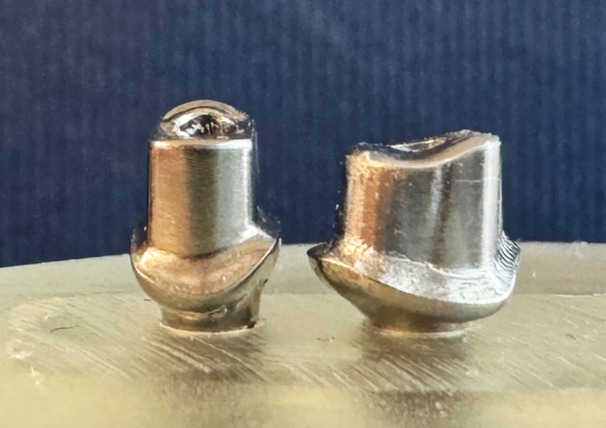
Premolar and molar custom designed abutment.

To design the patient‐specific custom abutments, implant analogues were embedded in a cast of a training model with a missing molar and premolar in separate regions of the maxilla. After scanning, the dental laboratory designed the reconstruction and ordered the corresponding abutments using the workflow for individually milled abutments of both manufacturers. The same designs (Figure 1) were used to order all molar (6.2 × 6.2 × 4.5 mm) and all premolar (5.7 × 3.8 × 4.5 mm) abutments.

Stock abutments were ordered through regular procedures and used without modification. Custom titanium test caps were designed for each type of abutment, with the correct height and surface curvature to adhere to the ISO 14801 protocol.

The 60 milled titanium custom abutments were equally distributed between molars and premolars and between nonoriginal and original, and were checked by a lab technician to ensure a proper fit of the test cap.

Abutments were randomly assigned to the testing pucks and implants and placed on the implants. The screws were tightened to the manufacturer's recommended torque value (20Ncm) for the implant system using a single ratchet (ZimVie Low Torque Indicating Ratchet Wrench, ZimVie Dental). The calibration of the ratchet was conducted following the procedure outlined by Stroosnijder (Stroosnijder et al., 2016). The torque values were checked after 10 min to ensure proper screw tightening, as recommended in previous studies (Rizvi et al., 2022).

Abutment screw channels were filled with polytetrafluoroethylene tape before cementing the titanium test cap using a self‐adhesive resin cement (Panavia SA Cement Universal, Kuraray Noritake Dental Inc.). Testing was carried out after allowing sufficient time for the cement to harden according to manufacturer's protocol.

### 2.3 Thermomechanical aging, load‐to‐failure test, and failure mode analysis

**Figure 2:**
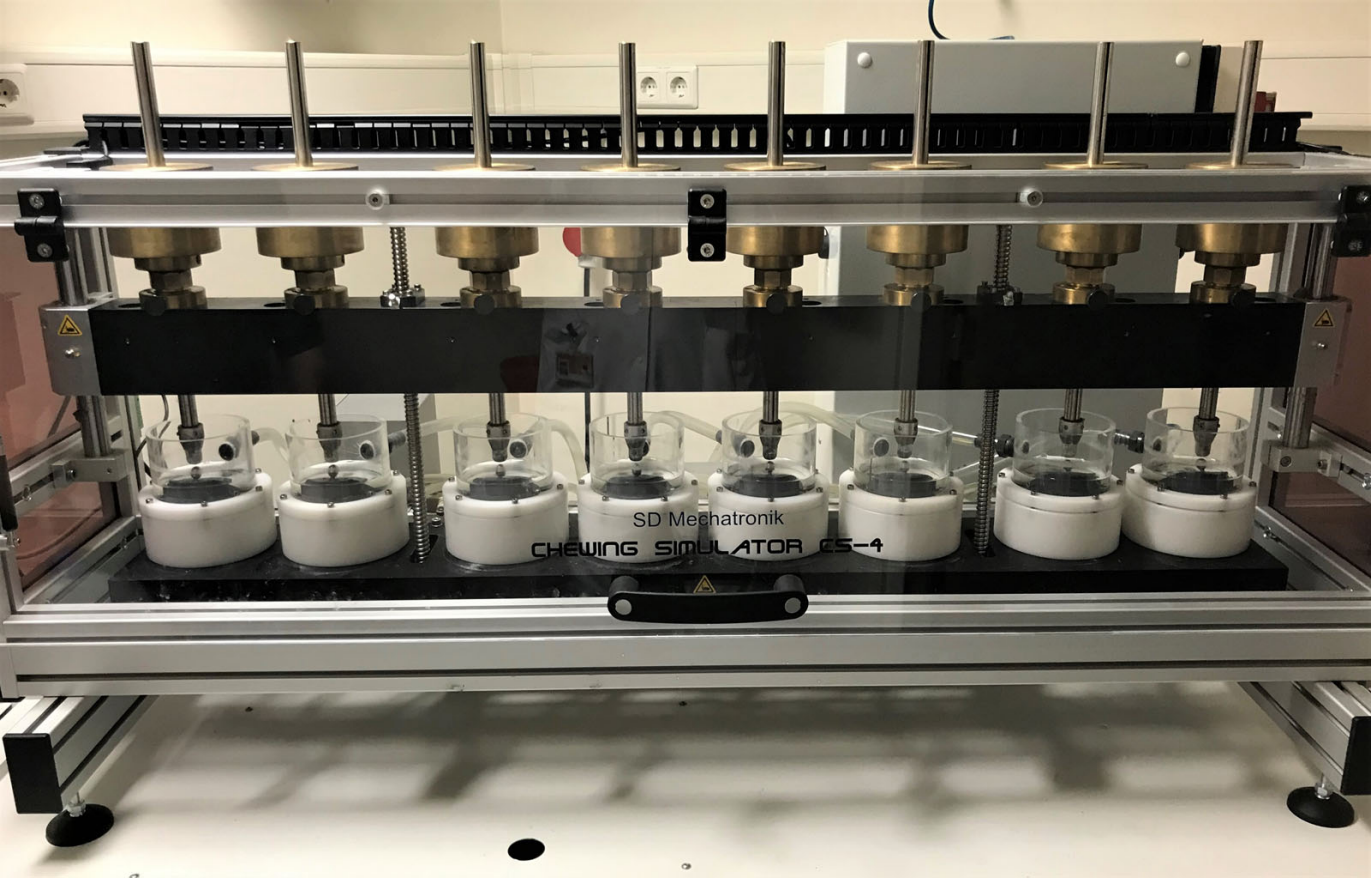
Overview of the chewing simulator and thermocycler.

Samples of each type were randomly assigned to test protocols and evenly distributed across test cycles. From each of the six groups, five samples (*n* = 30) were kept as control. The remaining samples (6 groups × 10 samples) were thermo‐mechanically aged using a chewing simulator (SD Mechatronik CS‐4.8 Chewing Simulator) at 1.7 Hz with an axial load of 50N under a 30° angle provided by a ceramic sphere as antagonist. Testing was done with simultaneous thermal cycling of 5–55°C, with a dwelling time of 30 s. From each group, five samples (*n* = 30) were aged to simulate 5 years of clinical function (1.2 × 10^6^ chewing cycles and 8000 thermo‐cycles), while the other five samples (*n* = 30) were aged to simulate 10 years of service (2.4 × 10^6^ chewing cycles and 16.000 thermo‐cycles). This procedure is illustrated in Figure 2 and follows testing protocols described in previous studies (Alonso‐Pérez et al., 2021; Rosentritt et al., 2009; Sailer et al., 2018).

**Figure 3:**
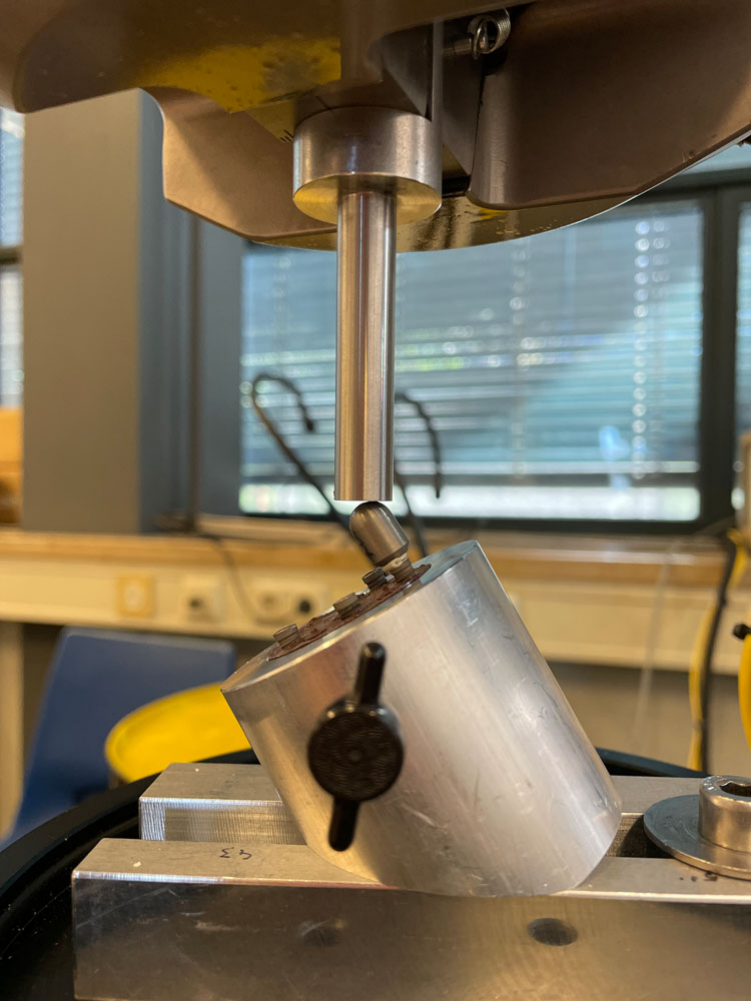
A sample being tested to failure with the test cap cemented on a stock abutment.

All implant‐abutment combinations (*n* = 90) were then tested to failure following a procedure described in the ISO 14801 protocol, using a universal testing machine (MTS‐810, MTS Systems) under a 30° angle at 1 mm/min with a hardened, stainless steel 5 mm sphere as antagonist, as illustrated in Figure 3.

Failure mode analysis after compressive load to failure testing was performed using optical microscopy (Zeiss Extaro 300; Carl Zeiss Meditec) at up to ×5 magnification.

For each specimen the failure mode was classified according to the implant component where the failure and/or fracture occurred: fracture of the implant or abutment, bending, creasing of deformation of the implant or the abutment, loosening of the connection screw and fracture of the connection screw. Samples in which more than three modes of failure occurred, were also marked as having mixed modes of failure.

Scanning electric microscopy (SEM; Zeiss Supra55 Atlas, VP FE‐SEM; Carl‐Zeiss Microscopy) was used to survey questionable failures and to analyze representative samples from each group and failure mode.

### 2.4 Statistical analysis

Descriptive statistics were utilized to present the maximum force, bending moments, events, and reasons for failure. Normality and homogeneity of variance assumptions were verified using Levene's test. A two‐way ANOVA test was employed to analyze the load at failure as the dependent variable, with aging (0/5/10‐year equivalent), manufacturer (nonoriginal/original), or design (stock/molar/premolar) as independent variables. Post hoc tests for multiple comparisons were carried out using the Tukey correction. Fisher's Exact or Fisher‐Freeman‐Halton Exact tests were used to analyze the results of failure mode classification. Statistical analysis was performed using IBM SPSS Statistics 28.0 (IBM Corporation), with a statistical significance level set at *α* = .05 for all tests.

## 3 RESULTS

No failures occurred during the thermo‐mechanical aging process. Tables 2 and 3 presents data on events, mechanical strength, and failure mode for each group. The mean mechanical strength across all groups was 1009N ± 171 and varied between 735N and 1504N for the 90 samples. The results of the statistical analysis are shown in Table 4. With all samples from all aging regimes combined, significant differences were found between nonoriginal and original abutments, both overall (−230N ± 27.1, *p* < .001) and within each abutment type favoring original components (all *p* = .000). Furthermore, original components were significantly stronger than nonoriginal components within most aging groups, as presented in Table 4. Customized abutments were found to be significantly stronger than stock abutments (80N ± 37.8, *p* = .037) when combining both original and nonoriginal groups. Thus, the hypothesis that there is no significant negative effect of nonoriginal or stock components on mechanical strength, was rejected.

**Table 2:** Overview of results (custom abutments).

Abutment group	Nonoriginal			Original			Nonoriginal			Original		
	Custom molar			Custom molar			Custom premolar			Custom premolar		
Thermomechanical aging protocol	No ageing	5‐year	10‐year	No ageing	5‐year	10‐year	No ageing	5‐year	10‐year	No ageing	5‐year	10‐year
Sample size	5	5	5	5	5	5	5	5	5	5	5	5
Events during ageing	0	0	0	0	0	0	0	0	0	0	0	0
*Results of load‐to‐failure mechanical strength test (N)*												
Mean maximum force ± SD	972 ± 105.8	896 ± 104.9	1002 ± 196.1	1229 ± 23.3	1034 ± 32.8	1184 ± 96.1	847 ± 70.8	897 ± 76.7	851 ± 93.1	1130 ± 181.4	1248 ± 148.9	1141 ± 196.6
Minimum	875	822	735	1204	998	1102	777	823	775	920	1139	942
Maximum	1132	1074	1270	1258	1086	1345	964	1005	1005	1292	1504	1373
*Failure information (n* = *)*												
Implant fracture	0	0	0	1	0	0	0	0	0	0	0	0
Implant bended/creased/deformed	5	5	5	5	5	5	5	5	5	5	5	5
Abutment fractured	0	0	0	1	2	0	0	0	0	0	1	1
Abutment bended/creased/deformed	1	3	1	5	0	4	4	1	4	3	2	3
Screw loosening	0	0	0	0	0	0	0	0	0	0	0	0
Screw fractured	5	5	5	0	1	1	4	5	5	2	3	2
*Mixed (≥3 failures)*	*1*	*3*	*1*	*3*	*0*	*0*	*3*	*1*	*4*	*1*	*2*	*1*

**Table 3:** Overview of results (stock abutments).

Abutment group	Nonoriginal			Original		
	Stock abutment			Stock abutment		
Thermomechanical aging protocol	No ageing	5‐year	10‐year	No ageing	5‐year	10‐year
Sample size	5	5	5	5	5	5
Events during ageing	0	0	0	0	0	0
*Results of fracture strength test (N)*						
Mean maximum force ± SD	885 ± 103.0	838 ± 71.9	861 ± 52.8	1148 ± 121.8	1021 ± 78.0	981 ± 135.1
Minimum	778	736	791	1016	955	831
Maximum	1041	931	906	1262	1152	1189
*Failure information (n* = *)*						
Implant fracture	0	0	0	1	1	1
Implant bended/creased/deformed	5	5	5	5	5	5
Abutment fractured	0	0	1	0	3	1
Abutment bended/creased/deformed	4	2	4	5	3	4
Screw loosening	0	0	0	0	0	0
Screw fractured	5	5	5	0	0	0
*Mixed (≥3 failures)*	*4*	*2*	*4*	*1*	*3*	*1*

**Table 4:** Comparison of fracture strength between nonoriginal and original abutments of three different abutment designs with *p* Values adjusted for multiple comparisons by Tukey's method.

			Nonoriginal	Original	Mean difference	Standard error	*p* Value	95% confidence interval	
	Interval	*n* =	Mechanical strength (*N*)	Mechanical strength (*N*)				Lower bound	Upper bound
Custom molar design abutment	No ageing	5	972 ± 105.8	1229 ± 23.3	−257.00	73.413	.069	−522.64	8.64
	5 years	5	896 ± 104.9	1034 ± 32.8	−137.80	73.413	.911	−403.44	127.84
	10 years	5	1002 ± 196.1	1184 ± 96.0	−182.00	73.413	.553	−447.64	83.64
	*Overall*	15	*956* ± *139.5*	*1149* ± *102.9*	−192.27	42.385	.000	−315.36	−68.17
Custom premolar design abutment	No ageing	5	847 ± 70.8	1130 ± 181.4	−283.80	73.413	.024	−549.44	−18.16
	5 years	5	897 ± 76.7	1248 ± 148.9	−351.20	73.413	.001	−616.84	−85.56
	10 years	5	851 ± 93.1	1141 ± 196.6	−290.60	73.413	.018	−556.24	−24.96
	*Overall*	15	*865* ± *78.4*	*1173* ± *172.6*	−308.53	42.385	.000	−432.63	−184.44
Combined custom abutment	No ageing	10	909 ± 107.6	1180 ± 132.5	−270.40	55.708	<.001	−458.10	−82.70
	5 years	10	896 ± 86.6	1141 ± 152.0	−244.50	55.708	.002	−432.20	−56.80
	10 years	10	926 ± 165.2	1163 ± 147.6	−236.30	55.708	.003	−424.00	−48.60
	*Overall*	30	*911* ± *107.6*	*1161* ± *140.2*	−250.40	33.760	<.001	−317.98	−182.82
Stock abutment	No ageing	5	885 ± 103.0	1148 ± 121.8	−263.00	73.413	.055	−528.64	2.64
	5 years	5	838 ± 71.9	1021 ± 77.9	−182.20	73.413	.551	−447.84	83.44
	10 years	5	861 ± 52.8	981 ± 135.0	−120.60	73.413	.971	−386.24	145.04
	*Overall*	15	*861* ± *75.5*	*1050* ± *128.9*	−188.60	42.385	.000	−312.70	−65.50
Overall—All designs combined	No ageing	15	901 ± 103.0	1169 ± 125.6	−267.93	47.122	<.001	−405.37	−130.5
	5 years	15	877 ± 84.3	1101 ± 141.5	−223.73	47.122	<.001	−361.17	−86.3
	10 years	15	905 ± 139.2	1102 ± 164.4	−197.73	47.122	<.001	−335.17	−60.3
	*Overall*	45	*895* ± *109.3*	*1124* ± *145.0*	−229.80	27.075	<.001	−283.61	−176.00
Overall molar—Premolar (*n* = 30)					33.73	29.971	.502	−37.99	105.46
Overall molar—Stock (*n* = 30)					97.03	29.971	.005	25.31	168.76
Overall premolar—Stock (*n* = 30)					63.30	29.971	.094	−8.42	135.02
Overall custom—Stock (*n* = 60/30)					80.18	37.772	.037	5.10	155.23

The study found that the aging regime imposed did not have a significant effect on failure load, while the type of abutment and manufacturer did show significant effects (aging: F(2,72) = 1.248, *p* = .293, *ω* = .034, abutment‐type: F(2,72) = 5.403, *p* = .007, *ω* = .131, manufacturer: F(1,72) = 88.185, *p* = .000, *ω* = .551), favoring custom designed and original abutments respectively. There was no significant interaction between aging and manufacturer with respect to failure load (F(2,72) = 0.701, *p* = .499, *ω* = .019), but the effect of aging differed significantly across the types of abutments (F(4,72) = 3.372, *p* = .014, *ω* = .158).

Information on the failure mode has been described in Tables 2 and 3. The most common failures were fracture of the implant and bending of the abutment or screw. Nonoriginal components exhibited significantly more modes of failure per sample (*p* = .006; two‐sided). Fracture of the connecting screw was significantly more common in nonoriginal abutments (*p* < .001; two‐sided). In original abutments, fracture of the abutment was more common (*p* = .015). The failure modes did neither differ significantly between aged groups nor between custom and stock abutments.

**Figure 4:**
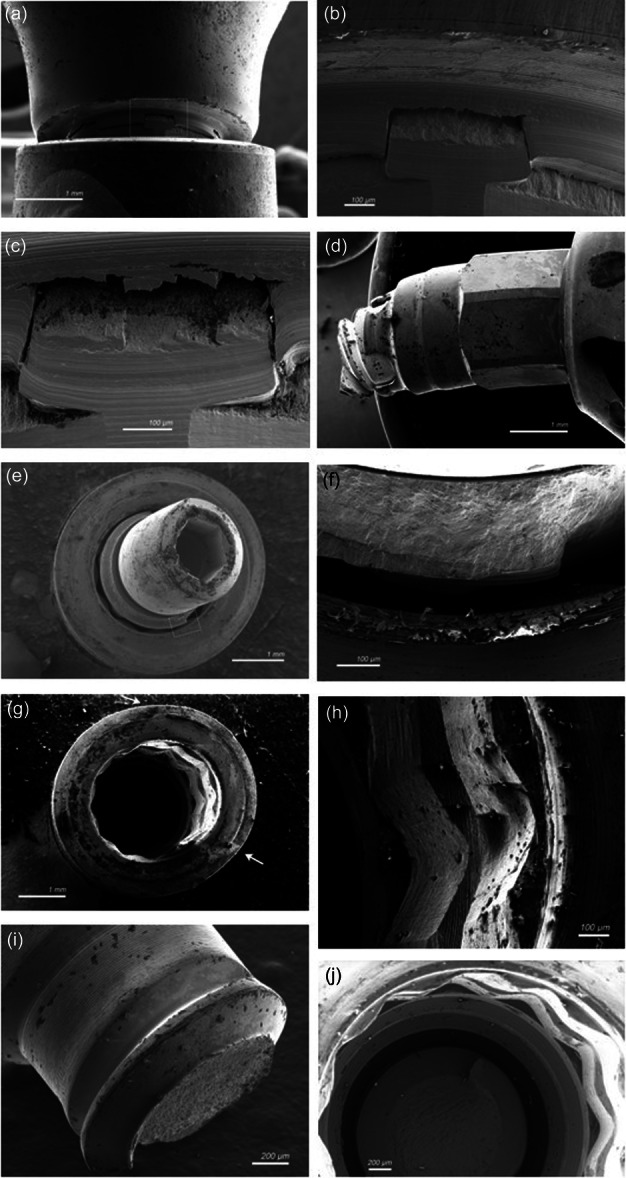
Representative SEM images of the various failure modes and abutments. (a) Fracture of a custom nonoriginal abutment (b) detail of (a) at indicated region/(c) fracture of a custom original abutment/(d) fractured screw and deformation of abutment in original custom abutment/(e) complete fracture of a custom original abutment/(f) detail of (e) at indicated region/(g) deformation of an implant/(h) detail of deformed implant with fracture forming/(i) fractured screw on abutment side, nonoriginal stock abutment/(j) fractured screw in‐situ in implant, nonoriginal stock abutment.

Representative SEM images from the groups and failure modes are shown in Figure 4. Microscopy and SEM evaluation of the failures revealed that abutment‐deformation and fracture patterns were similar across the various types and manufacturers (Figure 4a–f). The frequently observed implant deformation was found to propagate from edges in the internal connection (Figure 4g). Fractures of implants were less frequently observed, but were found to originate from the same locations where reduced wall thickness is present (Figure 4h). Broken screws all fractured at the point in the first or second thread with the smallest diameter and at the point of exit from the abutment (Figure 4d,i,j).

## 4 DISCUSSION

The aim of this in vitro study was to determine the effect of selecting stock or custom abutments from original or nonoriginal suppliers on the mechanical strength and failure modes of the implant‐abutment complex, aiding in selecting the optimal restorative solution, by using thermomechanical aging.

Overall, original abutments and screws were found to show a significantly higher mechanical strength compared to nonoriginal components in all groups. Custom fabricated abutments exhibited higher mechanical strength than stock abutments. The effect of the type of custom design was not significant and the mechanical strength was not significantly influenced by the thermo‐mechanical aging imposed in this study.

The results of the present study are in line with a recent systematic review on the use of nonoriginal implant components by Rizvi et al., although in that review only a very limited amount of studies was identified that matched the scope of the present study (Rizvi et al., 2022). Overall, nonoriginal abutments with an internal connection were found to be less compatible to the implant systems than original abutments, but this conclusion is based on only two articles (Alonso‐Pérez et al., 2018; Karl & Irastorza‐Landa, 2018). Other articles in the review either describe external connection, where the difference between original and compatible was found to be less distinct. Furthermore, the review reported on the use of original components on platforms of other manufacturers or systems, with various degrees of success.

Recent publications on the theme of nonoriginal implant abutments also seem to point in the direction of more favorable results for original components (Alonso‐Pérez et al., 2022; Berberi et al., 2022; Jarman et al., 2017). However, much of that research was performed, at least in part, on zirconia abutments. Original components generally outperformed the aftermarket varieties. Nevertheless, because of the limited strength reported for zirconia compared to titanium, the margin for error is significantly smaller, especially in more demanding indication, such as the posterior region.

Various explanations have been suggested to account for the difference in results for nonoriginal components. Most revolve around the degree of misfit. It is suggested that more misfit between implant and abutment leads to a higher degree of complications, such as screw loosening or screw fracture, especially after cyclic loading and fatigue testing (Alonso‐Pérez et al., 2022; Berberi et al., 2022; Fokas et al., 2019; Karl & Irastorza‐Landa, 2018; Karl & Taylor, 2016). This would entail that testing nonoriginal components from other suppliers, especially suppliers with less stringent quality control and design specifications, could lead to a larger distinction between original and nonoriginal components.

In the present study the thermomechanical aging did not show a significant effect of thermocycling on the mechanical strength. This contrasts with a recent systematic review on in vitro testing of implant abutments, stating that regardless of the manufacturer, increased number of cyclic loading decreased the mechanical strength of the implant components (Coray et al., 2016). This conclusion is, however, based on a limited number of studies since only three studies reported on mechanical strength without aging and these three studies used more loading force: 100–718N compared to 50N in the present study (Coray et al., 2016). Other previous research found that more effect of aging can be expected when thermomechanical aging is done with a higher load (Dittmer, et al., 2012; Strub & Gerds, 2003).

The sample size was chosen in accordance with previous studies and the ISO 14801 protocol, indicating at least three samples per force group. In the present study, 5 samples in each design or manufacturer were used as control and 10 samples as aged test. Since the influence of the duration of thermomechanical aging on strength was found to be not significant, an analysis of all 15 samples combined was also performed to increase the sample size for between‐group comparison.

Depending on the type of food and the amount of chewing cycles, normal chewing forces are reported to be around 50N, although initial chewing force may be higher (Schindler et al., 1998). The average reported maximum bite force exerted at the molar region ranges between 392N ± 150 and 720N (range: 244–1243) for patients with full dentition (Gibbs et al., 2002; Zivko‐Babić et al., 2002). Normal masticatory forces are lower—varying between 17N and 450N (Morneburg & Pröschel, 2002; Schindler et al., 1998), while patients with bruxism may exert involuntary forces of up to 1100N (Van Der Bilt, 2011).

The mechanical strength of the final reconstruction is influenced by several factors, and one of them is the screw that is used to connect the implant and abument. In all samples the screw provided with the abutment was used. It has been observed that screw fracture occurred more frequently in samples with nonoriginal abutments, while screw deformation was common across most samples. These findings are consistent with a previous study (Yilmaz et al., 2015). From a clinical perspective, a screw fracture as the primary mode of failure can be advantageous. The location of the fracture would allow for removal of the fragment and replacement of the screw.

Previous research has also shown that a solid lubricant, such as a layer of gold or diamond‐like carbon, can enhance the performance of titanium screws with threads at the end, which are commonly used in abutments and also with the original abutments used in the present study. This enhancement in performance has been linked to an increased resistance to the implant‐abutment‐complex. Therefore, original abutments with titanium screws coated with solid lubricants can be expected to perform significantly better than nonoriginal alternatives (Prado et al., 2014).

Examining the failure mode in mechanical testing of dental implants yields valuable information concerning the implant's mechanical performance under specific loading conditions. This information can be used to develop more efficient implant systems that can better withstand the stresses encountered in a clinical environment. Moreover, it can help detect potential problems associated with a particular implant design, enabling necessary modifications before clinical use. Finally, it can offer insights into the material characteristics of the implant and the adjacent bone tissue, enabling optimization of the design and enhancement of implant durability. Overall, incorporating both data types (load to failure and failure mode) allows for a comprehensive assessment of the implant's mechanical behavior, providing information on the implant's load‐bearing capacity, the particular failure modes, and the underlying causes of failure (Bordin et al., 2018; Delben et al., 2014; Dittmer, et al., 2011; Magne et al., 2011).

In the present study testing was performed with a protocol based on worst‐case testing. Several factors, mainly the amount of simulated bone loss (3 mm) and the angulation (30°), aid in obtaining failure results, but might make the test less clinically relevant. This is further illustrated by the use of a test cap instead of a restoration. And while this allows for a more standardized test, this too might have an effect on the outcome. A restoration adds length to the implant‐abutment‐complex and thus increases the bending moment, putting more strain on the abutment connection (Mühlemann et al., 2014). A recent publication by Alonso‐Pérez using a similar setup and components to the present study, but with anatomically designed veneered reconstructions and cyclic fatigue failure testing, found that the implant‐abutment‐complex failed under cyclic loading without any fractures or chipping of the restorations. This suggests that the weak point in this mode of testing is in the metal components rather than the ceramics (Alonso‐Pérez et al., 2022).

The present study tested the samples under oblique loading, which was done at a 30° angle, and with 3 mm of bone loss. The results showed that the mechanical strength ranged from 735 to 1504N under these simulated worst‐case conditions. However, it is important to note that the clinical performance of the reconstructions under occlusal load is likely to be better than the reported load at failure after oblique loading. This is because lateral physiological chewing forces are typically lower than axial forces (Koolstra & van Eijden, 1992).

The protocol used in this current study follows the protocol presented in various other publications using the same equipment, making it possible to compare results (Alonso‐Pérez et al., 2021; Rosentritt et al., 2009; Sailer et al., 2018). On the other hand, some of the previously mentioned drawbacks of this protocol are also shared with other publications. The ISO 14801 protocol for testing implant components has been updated in 2016, but so far publications with this new protocol are difficult to find. The new protocol, compared to the 2007 version, changes the loading pattern to more dynamic loading and requires changes to the equipment to better counter lateral forces. For this publication, the existing test setup and protocol was used, based on the review of Coray et al. indicating that any form of fatigue loading would decrease strength of implant components (Coray et al., 2016).

Nevertheless, a recommendation can be made, to all researchers involved in laboratory testing of implant components, to look into the benefits of the new ISO 14801:2016 protocol.

When evaluating failure modes, different magnification levels are important—from naked eye view to scanning electron microscopy. Sufficient magnification can reveal the finer details of the implant and its component parts. Using all levels of magnification allows for a more complete analysis of the fracture surface. At the micro‐scale, information on the microstructural composition of the material can be collected, while macro‐scale analysis can reveal the overall fracture characteristics and surface features. By combining the two, a comprehensive picture of the fracture can be obtained. This allows for a more accurate diagnosis of the cause of the fracture and helps to identify potential corrective measures that can be taken. In a clinical context, it is crucial to recognize that several of the less obvious failures observed in this study were only revealed under microscopic examination with higher magnification or even using SEM. This highlights a potential for a false sense of repairability in clinical cases and thus the start of a new failure (Naves et al., 2020).

While the abutments in this study exhibited a mean mechanical strength of 1009N ± 171, well above the reported average maximum voluntary axial bite force (392–720N), there is still a possibility that in worst‐case scenarios, the physiological bite force may exceed the strength of the components with the lowest mechanical strength. Therefore, challenging clinical situations require a more careful selection of components, especially with the broad range of nonoriginal options available. Dentists are encouraged to make an informed decision when choosing the components for implant restorations, as these choices can have a significant impact on the strength of the reconstruction. Furthermore, randomized controlled clinical trails would be needed to verify the findings of this study.

## 5 CONCLUSIONS

Within the limitations of this study original abutments and connecting screws exhibited a higher mechanical strength compared to the nonoriginal alternative, regardless of the amount of simulated clinical use. Similarly, custom abutments showed higher mechanical strength compared to stock abutments. However, mechanical strength in all abutments tested was higher than average chewing forces reported in the literature, thus components tested in this study can be expected to perform equally well in clinical situations without excessive forces.

## AUTHOR CONTRIBUTIONS

Study concept was designed by Christiaan W. P. Pol, Lucas Z. Naves, and Henny J. A. Meijer with support from Gerry M. Raghoebar and Marco S. Cune. Testing and analysis was performed by Christiaan W. P. Pol and Lucas Z. Naves. Manuscript was prepared by Christiaan W. P. Pol, Lucas Z. Naves, and Henny J.A. Meijer with support from Gerry M. Raghoebar and Marco S. Cune. All authors read and approved the final manuscript.

## CONFLICT OF INTEREST STATEMENT

The authors declare no conflict of interest.

## Data Availability

The data used and analyzed during the current study are available from the corresponding author on reasonable request.
